# Hearing Handicap among Hearing Impaired Adults: An Observational Study

**DOI:** 10.31729/jnma.9199

**Published:** 2025-08-31

**Authors:** Leison Maharjan, Bhawana Dangol, Ram Binay Yadav, Nishchal Devkota

**Affiliations:** 1 Department of Otolaryngology, and Head & Neck Surgery, Patan Academy of Health Sciences, Lalitpur, Nepal; 2 Department of Research and Development, National Open College, Lalitpur, Nepal

**Keywords:** *handicap*, *hearing handicap*, *hearing impairment*, *hearing loss*

## Abstract

**Introduction:**

Hearing impairment is a prevalent yet under-recognized condition in Nepal. Adults with hearing loss may experience stigma, social isolation, emotional distress, and reduced quality of life. Evidence from Nepal on the psychosocial impact of hearing impairment remains limited. This study aimed to assess hearing handicap among adults with hearing impairment.

**Methods:**

A hospital-based, cross-sectional descriptive study was conducted among 103 adults with hearing impairment attending an Ear, Nose, and Throat department of tertiary hospital in Nepal. Data were collected using the validated Nepali version of the Hearing Handicap Inventory for Adults - Screening version, along with socio-demographic and audiological profiles. Descriptive statistics were used for analysis.

**Results:**

Of 103 participants, the mean age was 45.38±20.30 (95% CI:41.46 -49.30) years. Audiological assessment revealed 69 (67.99%) had bilateral hearing impairment, 45 (43.69%) had mild hearing loss, and 42 (40.78%) had sensorineural hearing loss. Overall, 66 (64.08%) of participants reported hearing handicap, with 43 (41.75%) classified as mild-to-moderate and 23 (22.33%) as severe hearing loss. Perceived handicap was reported among 16 (80%) older adults aged 60-74 years and 35 (72.92%) males. Similarly, 4 (100%) people with profound hearing loss compared to 23 (51.11%) with mild hearing loss reported handicap. Likewise, 34 (80.95%) with sensorineural hearing loss and 57 (82.61%) with bilateral involvement reported handicap.

**Conclusions:**

Most adults with hearing impairment experience hearing-related handicap, influenced by the severity, type, and laterality of hearing loss. The majority of these individual have severe degree, sensorineural type and bilateral hearing impairment.

## INTRODUCTION

Hearing impairment is a growing health concern in low-income countries. By 2050, over 700 million people are expected to need hearing rehabilitation.^1^ Prevalence is higher in South Asia, 24 per 1,000 versus 3.5 per 1,000 in high-income countries.^2^ This disparity is linked to poverty, limited healthcare, infections, and environmental factors.^3^ The International Classification of Functioning, Disability and Health (ICF) framework notes that disability arises from both impairment and environmental conditions.^4^ WHO estimates unaddressed hearing loss costs the global economy nearly one trillion dollars annually in healthcare, educational support, and productivity losses.^15^ In Nepal, 0.38% of the population is reported to have hearing impairment due to chronic otitis media, vaccine-preventable infections, and nutritional deficiencies.^6-8^ This may be underreported due to stigma, poor awareness, and insufficient screening. Limited access to hearing health results in poor outcomes, while stigma further contributes to social exclusion.^7,9,10^

Studies have shown inconsistent perceived handicaps, ranging from 33.2% to 92.8%.^11-13^ This study aims to assess the hearing handicap among adults.

## METHODS

This hospital-based, cross-sectional descriptive study was conducted at Patan Academy of Health Sciences (PAHS), Lalitpur, Nepal, over two months (May-June 2025). Ethical approval was obtained from the PAHS Institutional Review Committee (drs2504222009), and written informed consent was obtained from all participants.

The sample size was calculated using Cochran’s formula (Z^2^ * p * (1 - p))/ e^2^, based on a 92.8% prevalence (p) of hearing handicap reported in a previous tertiary hospital study in Kathmandu.^13^ With a 95% confidence level (Z = 1.96) and a margin of error (e) of 5%, the calculated sample size was 103. Purposive sampling was used to recruit adults aged >18 years with unilateral or bilateral hearing impairment. Patients with known mental, cognitive, psychological, or physical comorbidities, hearing aid users, fluctuating hearing loss, chronic problematic tinnitus or vertigo, and hearing impairment of less than one month were excluded.

Audiological assessment (Pure Tone Audiometry) was performed by a single audiologist using Grason-Stadler (GSI) Audiostar, calibrated following the American National Standards Institute (ANSI). Audiometer calibration was conducted regularly and complied with the International Standards Organization Protocol 389 (1991). Hearing impairment classifications were based on the WHO 2021 grading system, based on audiometric measurements.^2^ The hearing threshold was defined as the minimum sound intensity that an ear can detect as an average of values at 500, 1000, 2000, and 4000 Hz in the better ear. Hearing was considered normal when the threshold was below 20 dB in the better ear. Mild hearing loss was defined as a threshold between 20 and <35 dB, moderate as 35 to <50 dB, moderately severe as 50 to <65 dB, severe as 65 to <80 dB, profound as 80 to <95 dB, and complete or total hearing loss (deafness) as 95 dB or greater, all measured in the better hearing ear. Unilateral hearing loss was characterized by a threshold of <20 dB in the better ear and ≥35 dB in the worse ear.^2^ Additionally, hearing loss types were classified based on air-bone gap values: conductive hearing loss was defined by an air-bone gap greater than 10 dB, sensorineural hearing loss by a gap of 10 dB or less, and mixed hearing loss as a combination of both conductive and sensorineural components.

Socio-demographic information was collected alongside the Nepali language validated Hearing Handicap Inventory for the Adults-Screening version (HHIA-S).^14^ HHIA-S is a self-reported questionnaire adapted from the Hearing Handicap Inventory for the Elderly-Screening version (HHIE-S), designed to assess the social, emotional, and psychosocial impact of hearing loss in adults.^15^ It comprises 10 items (five emotional, five social) rated on a three-point Likert scale. Total scores range from 0 to 40, with higher scores indicating greater psychosocial handicap. Scores were categorized as no handicap (0-9), mild-to-moderate (10-25), or severe (26-40). HHIA-S is suitable for clinical evaluation, rehabilitation assessment, and monitoring hearing aid outcomes.

Data was entered in Google Sheets and analyzed using Python. Descriptive statistics were used to summarize sociodemographic characteristics, audiological findings, and hearing handicap severity.

**Table 1:** Socio-demographic factors and degrees of handicap (n=103).

Socio-demographic factors	No Handicap n(%)	Handicap		Total n(%)
		Mild-Moderate n(%)	Severe n(%)	
Age				
18-29	15(14.60)	9(8.70)	6(5.80)	30(29.10)
30-44	9(8.70)	10(9.70)	3(2.90)	22(21.40)
45-59	7(6.80)	7(6.80)	8(7.80)	22(21.40)
60-74	4(3.90)	12(11.70)	4(3.90)	20(19.40)
75+	2(1.90)	5(4.90)	2(1.90)	9(8.70%)
Gender				
Male	13(12.60)	23(22.30)	12(11.70)	48(46.60)
Female	24(23.30)	20(19.40)	11(10.70)	55(53.40)
Marital status				
Unmarried	13(12.60)	9(8.70)	6(5.80)	28(27.20)
Married	24(23.30)	34(33.00)	17(16.50)	75(72.80)
Education				
Illiterate	3(2.90)	11(10.70)	7(6.80)	21(20.40)
Literate	34(33.00)	32(31.10)	16(15.50)	82(79.60)
Family structure				
Nuclear	5(4.90)	11(10.70)	9(8.70)	25(24.30)
Joint	32(31.10)	32(31.10)	14(13.60)	78(75.70)
Employment status				
Unemployed	27(26.20)	26(25.20)	18(17.50)	71(68.90)
Employed	10(9.70)	17(16.50)	5(4.90)	32(31.10)
Total	37(35.92%)	43(41.75)	23(22.33)	103(100)

## RESULTS

The study included 103 adults aged 18-87 years with a mean age of 45.38 ± 20.30 (95% CI 41.46 to 49.30) years. There were 30 (29.13%) participants aged 18-29 years, while 9 (8.74%) participants were above 75 years. There were 55 (53.40%) females and 48 (46.60%) males. Of the total participants, 75 (72.82%) were married, and 82 (79.61%) were literate. 78 (75.73%) individuals lived in joint families, and 71 (68.93%) individuals were unemployed.

Pure Tone Audiometry revealed mild hearing loss in 45 (43.69%) people, while 58 (56.31%) had moderate to profound hearing loss. Sensorineural hearing loss was observed in 42 (40.78%) individuals, conductive hearing loss in 38 (36.89%) individuals, and mixed hearing loss in 23 (22.33%) people. Bilateral impairment was present in 69 (67.99%) people.

Regarding the degree of perceived handicap, 37 (35.92%) individuals had no handicap, 43 (41.75%) individuals had mild to moderate handicap, and 23 (22.33%) individuals had severe handicap.

**Table 2:** Hearing impairment characteristics with degree of handicap (n=103).

Hearing impairment characteristics	No Handicap n(%)	Handicap		Total n(%)
		Mild-Moderate n(%)	Severe n(%)	
Degree of hearing impairment				
Mild	22(21.40)	18(17.50)	5(4.90)	45(43.70)
Moderate	7(6.80)	13(12.60)	6(5.80)	26(25.20)
Moderate-Severe	7(6.80)	8(7.80)	6(5.80)	21(20.40)
Severe	1(1.00)	1(1.00)	5(4.90)	7(6.80)
Profound	0	3(2.90)	1(1.00)	4 (3.90)
Type of hearing impairment				
Conductive	20(19.40)	14(13.60)	4(3.90)	38(36.90)
SNHL	8(7.80)	22(21.40)	12(11.70)	42(40.80)
Mixed	9(8.70)	7(6.80)	7(6.80)	23 (22.30)
Laterality of hearing impairment				
Unilateral	25(24.30)	7(6.80)	2(1.90)	34 (33.00%)
Bilateral	12(11.70)	36(35.00)	21(20.40)	69 (67.00%)

SNHL = Sensorineural hearing loss.

**Table 3:** Age-wise distribution of hearing handicap by type and degree of hearing loss (n=103).

Age group	Type of hearing loss^*^	No handicap n(%)	Handicap (Mild-moderate + Severe handicap) n(%)
18-29	Conductive	14 (73.68%)	5 (26.32%)
	Sensorineural	0 (0.00%)	9 (100.00%)
30-59	Conductive	6 (33.33%)	12 (66.67%)
	Sensorineural	4 (33.33%)	8 (66.67%)
60+	Conductive	0 (0.00%)	1 (100.00%)
	Sensorineural	4 (18.18%)	18 (81.82%)
Age group	Degree of hearing loss	No handicap	Handicap (Mild-moderate+ Severe handicap)
18-29	Mild (20-35 dB)	10 (66.67%)	5 (33.33%)
	Moderate+ Moderately severe (35-65 dB)	5 (41.67%)	7 (58.33%)
	Severe+ Profound (65+ dB)	0 (0.00%)	3 (100.00%)
30-59	Mild (20-35 dB)	10 (55.56%)	8 (44.44%)
	Moderate+ Moderately severe (35-65 dB)	6 (24.00%)	19 (76.00%)
	Severe+ Profound (65+ dB)	0 (0.00%)	1 (100.00%)
60+	Mild (20-35 dB)	2 (16.67%)	10 (83.33%)
	Moderate+ Moderately severe (35-65 dB)	4 (23.53%)	13 (76.47%)
	Severe+ Profound (65+ dB)	0 (0.00%)	0 (0.00%)

* Mixed hearing loss was excluded.

Of the total participants, 66 (64.08%) had perceived some degree (mild-moderate and severe) of handicap. Within socio-demographic categories, some degree (mild-moderate and severe) of handicap was reported among 16 (80%) older adults aged 60-74 years, 35 (72.92%) males, 51 (68%) married people, 18 (85.71%) illiterate people, 20 (80%) residents of nuclear families and 22 (68.75%) employed people. Similarly, 4 (100%) participants with profound hearing loss, 6 (85.71%) participants with severe hearing loss, and 23 (51.11%) participants with mild hearing loss had perceived some degree of handicap.

Additionally, 34 (80.95%) people with sensorineural hearing loss and 18 (47.37%) people with conductive hearing loss had perceived some degree of handicap. Furthermore, 57 (82.61%) people with bilateral involvement and 9 (26.47%) people with unilateral involvement had perceived some degree of handicap.

Among 18-29 years of age group, 5 (26.32%) people with conductive hearing loss handicap and 9 (100%) people with sensorineural hearing loss reported handicap. In adults over 60 years, 1 (100%) adult with conductive loss and 18 (81.82%) adults with sensorineural loss reported a handicap (Table 3).

In 18-29 years of age group, 5 (33.33%) people reported handicap with mild (20-35 dB) hearing loss loss, 7 (58.33%) people with moderate+moderately severe (35-65 dB) hearing loss, and 3 (100%) people with severe+profound (65+ dB) hearing loss. Among 30-59 years of age groups, 8 (44.44%) adults reported handicap with mild hearing loss, 19 (76.00%) people with moderate+ moderately severe hearing loss and 1 (100%) in the severe+ profound hearing loss. In adults over 60 years, 10 (83.33%) people reported handicap with mild hearing loss and 13 (76.47%) people with moderate+moderately severe hearing loss, with no Severe+Profound hearing loss case.

## DISCUSSION

The growing recognition of hearing impairment as a global health priority has led to increased attention from international bodies. The WHO’s World Report on Hearing 2021 emphasizes people-centered hearing care and introduces the H.E.A.R.I.N.G. framework (Hearing screening and intervention; Ear disease prevention and management; Addressing causes and their risk factors; Rehabilitation services; Improving access and affordability; Networking and support; and Generating evidence and promoting research).^1^ Hearing impairment, as an invisible disability, can lead to social rejection and discrimination, with individuals often misjudged due to communication challenges.^10^

In this study, perceived hearing handicap (mild-moderate and severe combined) was more frequently reported among older adults (60-74 years), males, married individuals, those with lower literacy, residents of nuclear families, and employed participants. (Table 1) Regarding age, in our study young and middle-aged adults showed a progressive increase in handicap from mild to severe loss, whereas older adults reported substantial handicap even at mild and moderate levels. (Table 3) Evidence from Jordan and a review article also indicates that older adults are more likely to report hearing-related handicap and may experience distinct challenges compared to younger individuals. ^16, 17^

Regarding gender, some studies suggests that the psychosocial impact of hearing impairment may vary, with certain studies indicating greater vulnerability among women. ^18-20^ In contrast, studies from India and the USA have reported higher rates of hearing-related handicap in men. ^21,22^ Higher perceived handicap among married and employed participants in our study may reflect greater social and occupational communication demands, which make hearing difficulties more noticeable and impactful. Likewise, higher perceived handicap among illiterate individuals and those in nuclear families may reflect reduced access to coping strategies and limited social support, amplifying the impact of hearing loss on daily communication. However, Nawal et al. reported no association between hearing handicap and employment status, education, or income.^21^ Whereas a study among Hispanic populations found that enabling resources, such as higher income, were linked to lower odds of self-reported hearing handicap.^20^ Additionally, other research has suggested that higher educational attainment is associated with reduced reporting of hearing-related handicap.^23^

In our study, 64.08% of participants reported some degree of hearing handicap (n=43, 41.75% mild-moderate and n=23, 22.33% severe handicap combined) which is comparable to an Indian study reporting 69%.^19^ Aryal et al. found lower overall prevalence of 33.2% but a similar severity distribution.^11^ In contrast, Shrestha et al., using HHIE among elderly participants (≥60 years), reported 92.8% with a handicap, likely reflecting age-related vulnerability.^13^ The broader age range (18-87 years, mean 45.3) may have diluted severity patterns compared to elderly-only studies, where higher rates of severe handicap were observed.^13,19^ The substantial proportion of participants reporting no handicap (35.9%) despite measurable hearing loss may reflect cultural normalization of disability in Nepal, where stigma and traditional beliefs can suppress self-reporting. This is consistent with research indicating that cultural context influences perceptions of disability and health-seeking behavior, including studies among Hispanic populations and cross-cultural surveys in multiple countries.^20, 23^ Normalization of hearing loss may also delay intervention, as Lin et al. reported that each 25 dB increase in hearing loss is associated with 6.8 years of cognitive aging.^24^

Our findings indicate that greater severity of hearing loss was observed with higher reporting of handicap. (Table 3) This gradient relationship is consistent with previous studies reporting increased psychosocial and cognitive risks with worsening hearing loss.^11,13,19,25^ A meta-analysis by Loughrey et al. further confirmed this association, demonstrating strong consistency across populations.^26^ The type of hearing loss was also observed with handicap. In young adults (18-29 years), a substantially higher proportion of those with sensorineural hearing loss (n=9/9, 100.00%) reported a handicap compared to 26.32% (n=5/19) of those with conductive loss, indicating a greater functional impact of sensorineural impairment in this age group. (Table 3) Sensorineural hearing loss appears more strongly linked to handicap, likely reflecting its poorer prognosis and limited treatment options, as also noted by Aryal et al. and Diniz et al.^11,27^ Bilateral hearing loss was associated with greater difficulties compared to unilateral loss, consistent with findings from Iwasaki et al. and Nawal et al.^21,28^ This may result from reduced compensatory mechanisms, as Rutherford et al. described in relation to the increased cognitive load imposed by hearing loss.^29^ While some studies have reported no association between the duration ofhearing loss and handicap, the cumulative psychosocial impact remains an important consideration.^21^

Our findings indicate that hearing impairment characteristics-severity, type and laterality, appeared more closely related to handicap than literacy, household composition, or employment. This suggests that psychosocial interventions may be more effective if tailored to clinical hearing profiles rather than demographic categories. Although our study did not evaluate intervention outcomes, existing literature highlights the positive impact of hearing aids. For example, a UK-based study reported improved cognitive outcomes with hearing aid use, independent of social isolation and depression.^30^ Further longitudinal research in Nepal is warranted to assess intervention effects and to explore cultural factors that may mediate outcomes.

A key strength of our study is the use of the validated Nepali HHIA-S tool.^14^ Nonetheless, several limitations must be considered. Recall and response bias may have influenced selfreported data, while the urban hospital setting could introduce selection bias. Finally, the quantitative design may not fully capture the depth of psychosocial experiences, and the HHIA-S, while robust, focuses narrowly on hearing-related handicap rather than broader quality-of-life dimensions.

## CONCLUSIONS

Most adults with hearing impairment experience hearing-related handicap, influenced by the severity, type, and laterality of hearing loss. The majority of these individual have severe degree, sensorineural type and bilateral hearing impairment.

## Data Availability

The data are available from the corresponding author upon reasonable request.
